# The double-edged sword of becoming a caregiver: dynamic impact on four dimensions of well-being in Norway

**DOI:** 10.1186/s40359-024-01623-x

**Published:** 2024-03-04

**Authors:** Fredrik Methi, Ragnhild Bang Nes, Vegard Skirbekk, Thomas Hansen

**Affiliations:** 1 Department of Health Services, Norwegian Institute of Public Health, Postboks 222, Skøyen, N-0213 Oslo, Norway; 2 Department of Mental Health and Suicide, Norwegian Institute of Public Health, Oslo, Norway; 3 Promenta Research Center, University of Oslo, Oslo, Norway; 4 Department of Philosophy, Classics, and History of Arts and Ideas, University of Oslo, Oslo, Norway; 5 Center for Fertility and Health, Norwegian Institute of Public Health, Oslo, Norway; 6 Department of Psychology, University of Oslo, Oslo, Norway; 7 Oslo Metropolitan University, Oslo, Norway

**Keywords:** Caregiving, Psychosocial well-being, Loneliness, Norway, Longitudinal analysis

## Abstract

**Background:**

Becoming a caregiver can be a transformative journey with profound, multifaceted implications for well-being. However, existing research predominantly emphasizes the negative aspects of caregiving, paying less attention to the positive sides. This study aims to explore the impact of transitioning into a caregiving role on various well-being indicators, such as negative hedonic, positive hedonic, eudaimonic, and social well-being.

**Methods:**

We use Norwegian panel data (2019–2021) and employ a combination of nearest-neighbour matching and a difference-in-differences approach to analyse well-being trajectories among new caregivers (*n* = 304) and non-caregivers (*n* = 7822). We assess ten items capturing the dimensions of negative hedonic (anxiousness, sadness, and worriedness), positive hedonic (happiness and life satisfaction), eudaimonic (contributing to others’ happiness, engagement, and meaning), and social (strong social relations and loneliness) well-being.

**Results:**

Our results show a general increase in negative hedonic well-being and a decline in positive hedonic well-being for new caregivers. These impacts are larger for caregivers providing daily care, compared to those providing weekly and monthly care, and for those providing care inside rather than outside their own household. We observe only minor differences regarding gender and age. Interestingly, we also notice neutral or beneficial changes for eudaimonic aspects of well-being; of note, caregivers generally experience an increased sense of contributing to others’ happiness.

**Conclusion:**

Our study reveals that adopting a caregiving role often leads to significant psychosocial challenges, especially in intensive caregiving situations. However, it also uncovers potential positive influences on eudaimonic aspects of well-being. Future research should explore underlying explanatory mechanisms, to inform strategies that enhance caregivers’ well-being.

**Supplementary Information:**

The online version contains supplementary material available at 10.1186/s40359-024-01623-x.

## Introduction

Informal caregiving, defined as the provision of unpaid care and assistance to family, friends, or others with chronic illnesses, disabilities, or age-related needs, plays an essential role in national healthcare systems. In many countries, informal caregiving constitutes a substantial portion of the total care provided, with estimates suggesting that informal caregivers contribute to as much as 80% of all long-term care services [1]. As the global population ages and public healthcare systems face mounting pressures due to funding and personnel shortages, the reliance on informal caregivers will likely grow significantly in the future [2–3]. Against this background, it is crucial to examine the consequences of informal caregiving to better understand, support, and improve the well-being of caregivers and, by extension, their care recipients.

Stepping into the caregiving role can mark a transformative experience. With profound responsibility for others there will be less time and energy for self-care, paid labour, and leisure activities. Previous research on caregiving has predominantly focused on the negative aspects of caregiving, showing adverse impacts on caregiver burden, stress, and the risk of mental health issues [4–8]. This focus on adverse outcomes is justified given that caregiving can impinge upon critical factors associated with psychological distress and diminished well-being. For example, caregiving may lead to physical health ramifications such as chronic musculoskeletal injuries, arthritis, and other chronic illnesses [9], disrupted sleep, a general decline in self-rated health [5], and even mortality [10]. Social challenges are another significant aspect of informal caregiving. Caregivers often experience reduced social participation and increased social isolation and loneliness [11]. Moreover, caregivers frequently encounter difficulties in balancing their caregiving duties with other responsibilities, such as work, household tasks, and child-rearing, which can contribute to role strain and conflict as economic challenges [12–13].

Caregiving experiences and the associated distress are highly variable, reflecting a broad spectrum of challenges and responses that caregivers face. This variability can be understood through appraisal theory [14], which suggests that individuals’ perceptions of and reactions to caregiving roles significantly influence their emotional and psychological well-being. For instance, a caregiver who appraises their caregiving duties as a fulfilling responsibility may experience less distress compared to another who perceives the same duties as overwhelming and without support. Similarly, according to stress-coping theories of caregiving [15–16], the level of distress experienced by caregivers is shaped by both the demands of caregiving and the coping resources at their disposal. These resources encompass social support networks, formal assistance services, economic benefits, good health, and the reduction of role conflicts, such as those that occur between work and caregiving responsibilities. Consequently, individuals facing challenges such as limited social support, lower levels of education, simultaneous employment, or personal health issues are more likely to experience significant negative effects [16–17]. While these models and findings have been instrumental in illuminating the challenges faced by caregivers, they may have contributed to overlooking the potential positive effects of caregiving. Caregivers may feel a sense of meaningfulness and fulfilment, driven by the knowledge that their efforts are instrumental in improving the quality of life for their care recipients. The prosocial characteristics inherent in caregiving may also significantly augment a sense of mattering, a key factor in psychological well-being [18]. Generosity and contribution are for example key to Prilleltensky’s mattering theory, providing an experience of making a difference, being important, and feeling appreciated [18]. To develop a holistic understanding of the complex relationship between caregiving and well-being, more attention needs to be paid to multidimensional effects along the various dimensions of psychological well-being [19].

### Dimensions of well-being in the context of caregiving

Psychological well-being, also known as subjective well-being, includes both cognitive judgments and emotional responses. These two elements combine to form what is commonly referred to as ‘hedonic well-being’, an assessment of life satisfaction and happiness. The cognitive aspect, referred to as ‘cognitive well-being’, pertains to satisfaction with life overall or with specific life domains. The emotional dimension, termed ‘affective well-being’, refers to emotional experiences and differentiates between positive or pleasant feelings (such as joy, pride, and happiness) and negative or unpleasant emotions (like sadness, depression, and loneliness) [20]. Conceptually and empirically, these components are related yet distinct aspects of well-being, with partly unique both genetic and environmental underpinnings [21–22]. Additionally, the aspects of hedonic well-being can be grouped into positive and negative hedonic well-being. Positive hedonic well-being encompasses the elements of positive emotions, highlighting individuals’ experiences of life satisfaction and happiness. In contrast, negative hedonic well-being focuses on the presence of negative emotions and dissatisfaction, including feelings of sadness, anxiety, and worry. This dichotomy allows researchers and practitioners to distinctly measure and address the complex dimensions of well-being.

Because caregiving may have a multifaceted impact (e.g., emotional, structural, social, financial, health-related, existential), the effects of caregiving on psychosocial well-being could vary substantially depending on the well-being aspect under scrutiny and the caregiver’s other life circumstances [23]. Caregivers may for example experience emotional distress yet consider their lives highly meaningful and rewarding. Hence, caregiving research may benefit substantially from a more holistic study of psychological well-being and measures that are sensitive both to the day-to-day costs and the possible long-term or existential rewards of caregiving.

Rooted in ancient philosophy, the concept of eudaimonia, often associated with Aristotle, provides a valuable framework for exploring the positive dimensions of caregiving. Eudaimonia encompasses the pursuit of self-realization, personal growth, and the cultivation of virtues contributing to a meaningful and fulfilling life [24]. At its core, eudaimonic well-being involves engaging in challenging yet meaningful endeavours– particularly those demanding considerable effort, imbued with altruism, and oriented towards “the greater good” [24]. Caregiving has been identified as one such “worthwhile cause” [25]. In this context, the caregiving experience emerges as a dynamic and complex interplay of factors that may have negative implications for caregivers’ well-being while simultaneously incorporating positive aspects. The eudaimonic perspective on well-being, gaining significant influence in recent years (e.g [24–26])., underscores existential facets of well-being, emphasizing finding meaning, purpose in life, growth, and personal development.

Informal caregiving can foster stronger emotional bonds between caregivers and recipients, promoting mutual feelings of love, gratitude, and companionship [27]. Social well-being, here defined as the quality of social relationships and a sense of belonging, may both play a crucial role in the caregiving experience. Caregiving may also serve as a catalyst for fostering social well-being, through forming bond and connections with support groups, the care recipient and strengthening of social ties and resilience. For caregivers themselves, the experience of providing care can be a source of personal growth, meaningful achievement, improved self-esteem, and life satisfaction [5]. Quantitative studies show that situational variables such as the relationship between the carer and care recipient [28–29], the willingness to take on the caregiver role [30] and certain personality traits of the carer [31], such as agreeableness and extroversion, can be important moderators of positive effects. In addition, Pendergrass et al. [32] show that positive and negative effects of caregiving are not necessarily mutually exclusive, and that they both can co-exist. Qualitative interviews show that informal carers experience feelings of appreciation, increased affinity with the care recipient, personal growth, and satisfaction in their role as caregiver, and that these rewards can co-exist with high levels of stress [27, 33–34].

### Knowledge gaps

The literature has a number of other gaps that prevent a nuanced understanding of how caregiving may affect well-being. First, studies typically compare well-being levels of caregivers with non-caregivers through cross-sectional studies, not focusing on the longitudinal aspect and the profound change of *becoming* a caregiver (i.e., the role transition). When longitudinal studies exist, they often use panel data with three [35], four [36] or even five years [37] between the waves. This is problematic for two reasons. First, a typical caregiving career only lasts two to four years [37–39], which means that such studies will likely not capture the short-term effects of a transition into caregiving. Second, individuals typically revert to their baseline levels of well-being within a few years following an adverse change in life circumstances [40]. Hence, studies with long follow-up between the waves will likely not grasp what we are really interested in. A rare example of a study using more frequent data is Lacey et al. [41], in which the authors utilize data from the UK Household Longitudinal Study with one year between the waves.

Second, the existing literature on caregiving is disproportionally American, with limited European and Nordic evidence. The existing overarching European studies do not cover Norway [42–43]. While findings from these studies suggest that there are larger differences in well-being between caregivers and non-caregivers in Southern and Eastern-European countries with more traditional family and care style, they also highlight the need to look at countries where care responsibilities are shared between the public sector and the family. In Nordic countries such as Norway, the family and the public sector tend to share care responsibilities due to a robust welfare state, with informal caregivers mainly providing practical help and emotional support, while personal care, such as bathing, eating, and help with dressing are provided by the public sector [17]. Moreover, extensive employee rights often ensure that workplaces facilitate flexible work arrangements during a caregiving transition [44]. Hence, we would expect Norway to be considered a ‘best case’-scenario as the availability of formal care can buffer the negative effects of becoming a caregiver [42–43].

Finally, prior research rarely probes individual-level moderating effects. Caregivers are a diverse group, and the implications of caregiving may differ based on multiple individual and situational factors, including access to various resources. Grounded in stress-coping frameworks [16–17], caregiving distress relates to both caregiving intensity and coping resource availability. Specific factors, like older age or limited education, may exacerbate caregiving effects. Women, who commonly shoulder more caregiving than men [45], might experience exacerbated “strains and gains”. They may grapple with unique challenges like gendered societal expectations and restricted resource access, which could intensify negative caregiving effects [46]. With men’s increasing involvement in caregiving [47], understanding male caregiver experiences is becoming increasingly important, especially as gender roles evolve, evident in regions like the Nordic countries (e.g. [48])..

### This study

The main objective of this paper is to investigate the short-term impact of transitioning into a caregiving role on various dimensions of well-being, with a focus on exploring both the negative psychosocial effects and potential positive influences, as well as the eudaimonic and social aspects of well-being. We aim to extend the literature on well-being of caregivers in four main ways. First, we study short-term effects. This is to grasp the transitional effects of becoming a caregiver. Second, we study Norway, assumed to be a “best-case” country with a public sector providing formal care. Third, we stratify analyses by gender, age, and caregiving frequency, to examine intra-individual differences. And finally, we apply modern and popular quasi-experimental design from econometrics: the difference-in-differences method. This approach allows for a robust comparison even when there are varying baseline levels between groups. Not sufficiently accounting for baseline differences have been argued to be problematic in studies examining transitional effects of caregiving [49].

## Methods

### Data

We utilize data from the Norwegian Counties Public Health Survey (NCPHS), which is an online longitudinal study conducted among a probability sample of individuals aged 18 and above in Norway. The survey consists of four waves conducted in the counties of Agder and Nordland in Norway, as part of the covid-19 section of the NCPHS. The first wave was conducted from 23rd September to 18th October 2019, with a sample size of 28,015 and a response rate (RR) of 46% in Agder county. The same questionnaire was distributed from 27th January to 16th February 2020 in Nordland county, involving 24,199 participants with an RR of 47%. Subsequently, a random sample of 20,196 individuals from these counties was invited to participate in three follow-up surveys. The first follow-up (t2) was conducted from 4th to 18th June, 2020, with a sample size of 11,953 and an RR of 59%. The second follow-up (t3) took place from 18th November to 4th December 2020, involving 11,029 participants and an RR of 55%. Finally, the third follow-up (t4) was conducted from 6th to 20th December 2021, with a sample size of 10,220 and an RR of 52%. The initial waves (t1 and t2) were conducted prior to the pandemic, whilst the subsequent follow-up surveys (t3 and t4) took place during the pandemic.

### Independent variables

Caregiving was measured through a question formulated as follows “Did you, [in the given period], provide regular unpaid help or supervision to someone in need of help due to health problems or old age (e.g., housework, personal care, or supervision)? Please disregard work through a volunteer organization.” This question was only included in the questionnaire administered during the fourth and final round (t4). Hence caregiving in t1, t2, and t3 was measured retrospectively. Consequently, only the 10,220 participants who responded to the survey during the last round were eligible for inclusion in this study. Caregivers were identified as individuals who provided regular unpaid assistance or supervision to someone in need due to health issues or old age, engaging in such activities on a monthly basis or more [23]. For the purpose of this study, individuals who responded “no” in the initial wave(s) but indicated providing assistance on a monthly basis or more frequently in subsequent waves were classified as new caregivers. On the other hand, individuals who consistently responded “no” across all waves were classified as non-caregivers.

We included gender (male or female), county of residence (Agder or Nordland), education (compulsory, upper secondary, university < 4 years, university ≥ 4 years, or missing), partner status (married or registered partner, cohabiting, non-cohabiting partner, single, or missing), and age in pre-defined age groups (18–29, 30–39, 40–49, 50–59, 60–69, and 70+) as control variables. Both gender and county of residence remained consistent across all rounds in the study. For age, education, and partner status we used the value recorded at the initial survey (t1).

### Dependent variables

**Fig. 1:**
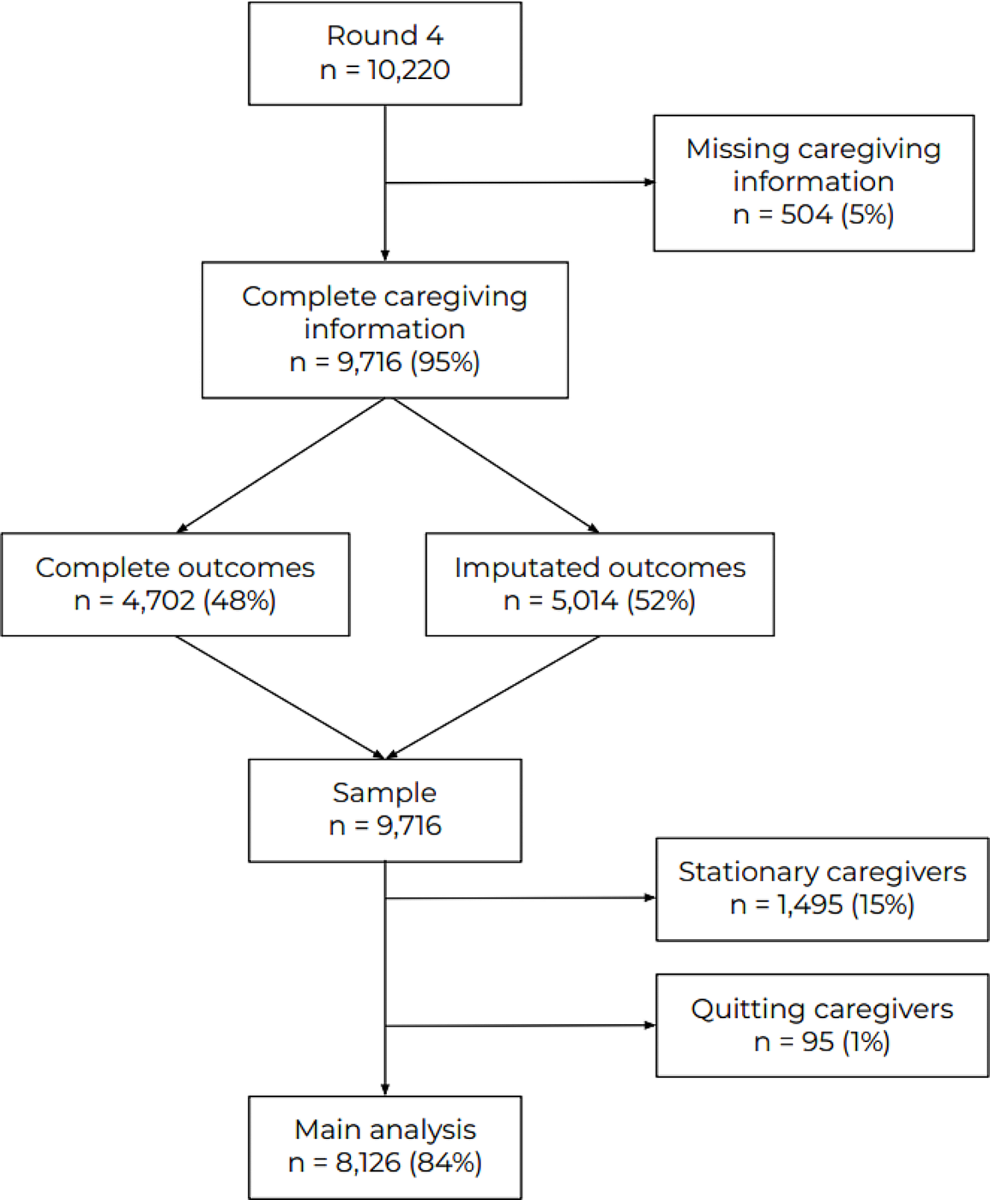
Flowchart of analytical sample

We employed ten variables to assess psychosocial well-being, each measured as a single-item variable on a scale ranging from 0 (“not at all”) to 10 (“very much”). Positive hedonic well-being included (1) happiness and (2) life satisfaction, and negative hedonic well-being included (3) anxiousness, (4) worriedness, and (5) sadness. The latter were initially measured from low (0) to high (10), but to ensure consistency in interpretation, we inverted the scales of the negative emotions, meaning that higher values on all items were associated with less illbeing/higher psychosocial well-being and lower values were associated with more illbeing/less psychosocial well-being. Eudaimonic well-being was measured through (6) contributing to other people’s happiness^1^, (7) meaning^2^, and (8) engagement in everyday life. Finally, social well-being included (9) social relations^3^, and (10) loneliness. The exact phrasing of each question is included in S-Table 1 and a correlation plot between the variables is included in S-Fig. 1.

### Statistical analyses

A common challenge analysing survey data is the case of missing data points. To address the issue of missing outcome values in our study, we used multiple imputation. Specifically, we generated five imputed datasets, each undergoing 50 iterations. The imputed values were selected using the predictive mean matching approach, which involves identifying the closest matches and randomly sampling one of these as the imputed value. The imputation procedure was conducted using the Mice package in R and laid the foundation for the main analysis. Additionally, as part of sensitivity analysis, we performed a complete case analysis.

**Fig. 2:**
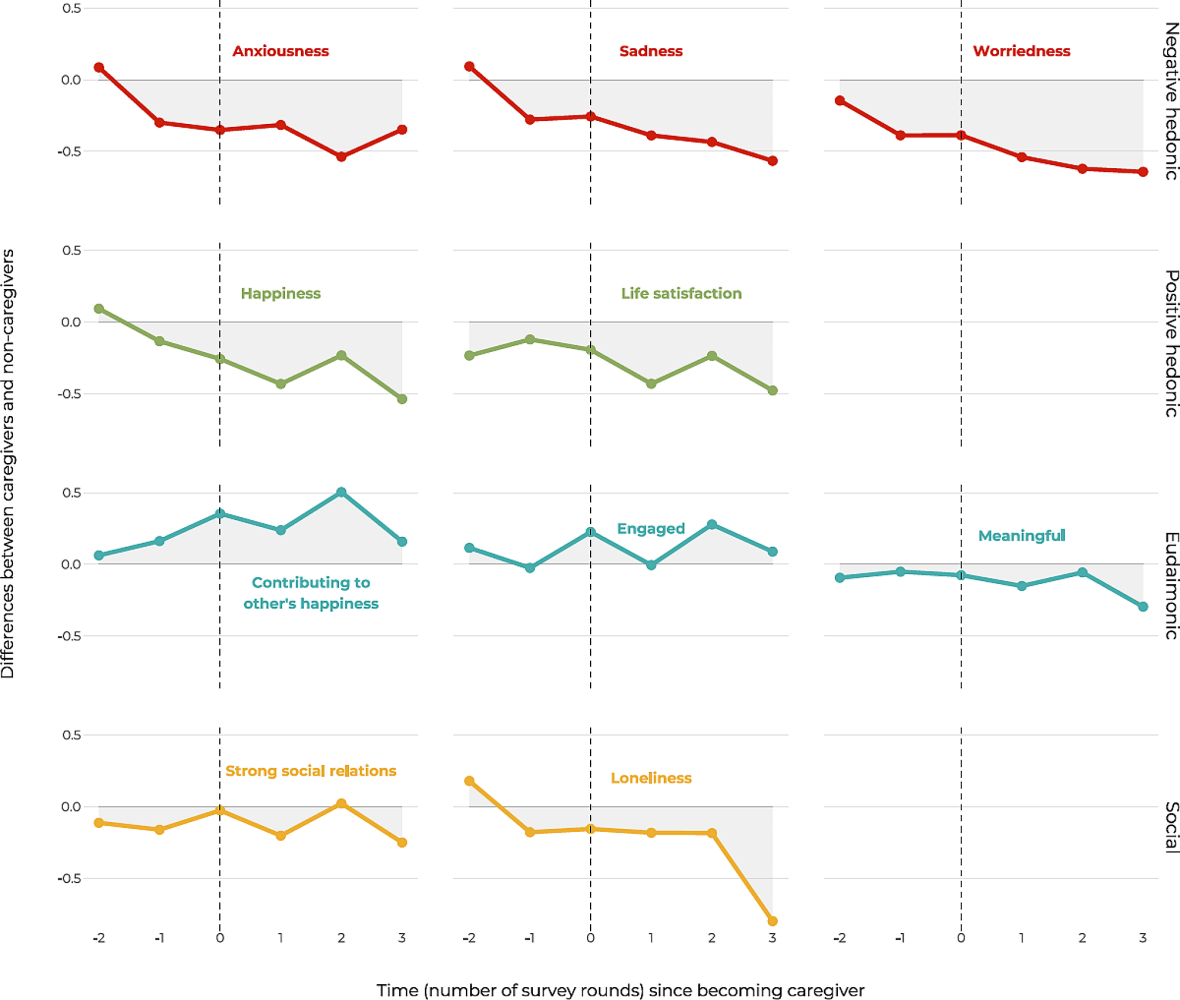
Differences in psychosocial well-being between new caregivers and non-caregivers

In the main analysis, our focus was on comparing new caregivers to those who remained non-caregivers throughout the study period, using the latter as a counterfactual group. To conduct this analysis, we excluded individuals who were consistently caregivers over the entire duration and individuals who ceased caregiving during the study period (included in sensitivity analysis). For new caregivers, we created a time variable that represented their time relative to becoming a caregiver. Specifically, we set time 0 as the timepoint before they reported to provide care, and time 1 as the first timepoint after becoming a caregiver, followed by time 2 for the subsequent timepoint, and so forth. To allocate time 0 to non-caregivers who did not have specific relative weeks, we applied a greedy matching technique which matched all non-caregivers to their nearest caregiving neighbour based on a propensity score taking into account age, marital status, education, gender and county of residence. If more than one caregiver were equally close, the match was chosen at random. Among new caregivers 33% transitioned at t2, 26% at t3, and 41% at t4. Among non-caregivers 37% were assigned a change at t2, 24% at t3, and 39% at t4 (S-Fig. 2).

To compare changes in well-being between new caregivers and non-caregivers, we employed a difference-in-differences (DiD) design [50]. The DiD design is a widely used quasi-experimental approach for estimating potential causal effects of interventions [51]. Technically, it involves running a regression that includes a dummy variable for the treatment group, a time dummy variable, and an interaction term between the treatment dummy and the time dummy. The key assumption of the DiD design is the presence of parallel trends among both groups during the period prior to the intervention, before the treatment group becomes caregivers. To address variations in the timing of switching caregiving status, we conducted sensitivity analyses using a modified DiD estimator accounting for the heterogeneous treatment effects proposed by Callaway and Sant’Anna [52].

Finally, to enhance our understanding of the factors influencing well-being and caregiving experiences, we conducted sub-group analyses stratified by gender, age, caregiving frequency, and whether the care recipient lived in the same residence as the caregiver.

## Results

### Descriptive statistics

Among the 10,220 participants in the fourth round of the NCPHS survey in Agder and Nordland, Norway, 9,716 (95%) had non-missing values on caregiving information and were included in our study population. Of these, 1,495 (15%) were characterised as caregivers throughout the whole period and 95 (1%) ceased caregiving during the period. Both groups were excluded from the main analysis (Fig. 1). This left us with 8,126 individuals in the main analysis.

Our study sample consisted of 7,822 individuals classified as non-caregivers, with 52.4% of them being female. Additionally, we included 304 new caregivers, among whom 54.7% were female. Table 1 provides an overview of the distribution of individuals who were included in our study. In terms of gender, new caregivers at the third (t3)) time point were more likely to be male, while at the second (t2) and fourth time points (t4), a larger proportion of new caregivers were female. In general, there wereno clear trends on partner status or education among compared with non-caregivers.

**Table 1:** Descriptive statistics

		Non-caregivers	New caregivers			Main sample
			t2	t3	t4	
Female		52.4%	58.0%	48.1%	49.6%	52.4%
Age						
	18–29	8.3%	5.0%	8.9%	7.2%	8.3%
	30–39	13.1%	13.0%	6.3%	15.2%	13.1%
	40–49	19.8%	20.0%	13.9%	16.8%	19.7%
	50–59	24.1%	26.0%	29.1%	27.2%	24.2%
	60–69	22.3%	18.0%	32.9%	22.4%	22.3%
	70+	12.4%	18.0%	8.9%	11.2%	12.4%
County						
	Agder	50.1%	70.0%	39.2%	57.6%	50.3%
	Nordland	49.9%	30.0%	60.8%	42.4%	49.7%
Partner						
	Single	18.0%	21.0%	24.1%	16.8%	18.1%
	Non-cohab. partner	5.9%	4.0%	8.9%	8.8%	6.0%
	Cohabiting partner	18.9%	14.0%	13.9%	20.0%	18.8%
	Married/reg. partner	56.9%	60.0%	53.1%	53.6%	56.9%
	Missing	0.2%	1.0%	0.0%	0.8%	0.2%
Education						
	Primary	10.7%	5.0%	8.9%	9.6%	10.7%
	Upper secondary	34.1%	32.0%	43.0%	34.4%	34.2%
	University < 4 years	24.8%	26.0%	22.8%	22.4%	24.8%
	University ≥ 4 years	30.0%	37.0%	25.3%	33.6%	30.1%
	Missing	0.3%	0.0%	0.0%	0.0%	0.3%
Caregiving						
	Monthly	-	55.0%	62.3%	51.2%	34.2%
	Weekly	-	33.3%	31.1%	36.0%	54.9%
	Daily	-	11.7%	6.6%	12.8%	11.0%
	Inside hh	-	25.0%	24.1%	29.6%	28.5%
	Outside hh	-	75.0%	75.9%	70.4%	71.5%
Total (N)		7 822	100	79	125	8 126

Note: hh means household

### Main analysis

**Fig. 3:**
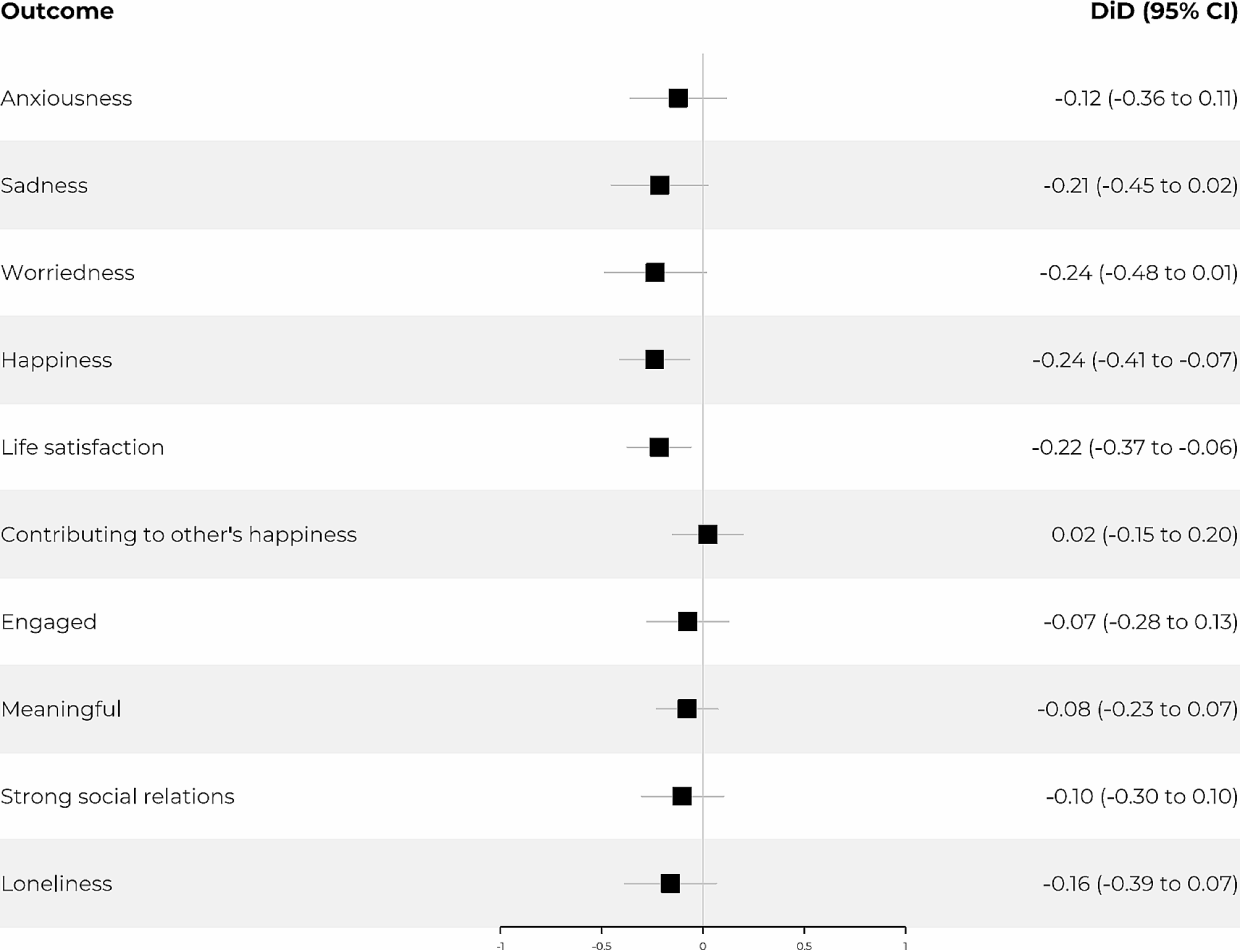
Forest plot. The plot visualise adjusted difference-in-differences (DiD) estimates with 95% confidence intervals for new caregivers compared to non-caregivers

Our main analysis consisted of two primary components. First, we conducted a comparison of the average levels of well-being indicators between new caregivers and non-caregivers, while adjusting for variables such as gender, age, education, partner status, and county (Fig. 2). We observed that the negative and positive hedonic measures all showed unfavourable trends for new caregivers compared to non-caregivers. Eudaimonic measures showed positive or little changes. Whereas the social measures showed little changes for social relations, but an adverse development for loneliness. Notably, we observed that for several indicators such as anxiousness, sadness and worriedness, there were pre-existing trends of adverse levels prior to individuals assuming the role of caregivers (Fig. 2). These trends were particularly prominent for the negative emotions and happiness, which all exhibited a steady unfavourable development throughout the study period starting before stepping into the caregiving role. Figure 2 shows the adjusted differences between new caregivers and non-caregivers. Separate trajectories are included in S-Fig. 3.

In the second aspect of our analysis, we acknowledged that scores on certain indicators were already adverse prior to becoming a caregiver. To account for this, we calculated DiD estimates. A positive coefficient in the DiD analysis suggests that becoming a caregiver had a positive impact on the outcome variable, while a negative coefficient indicates a negative effect. By employing this approach, we aimed to capture the specific effects of the caregiving intervention on the outcome measures, accounting for any pre-existing differences.

The DiD estimates, which are presented in a forest plot in Fig. 3 and in Table 2, reveal important findings regarding the impact of assuming the caregiving role. Specifically, the estimates indicate that new caregivers experienced a significant decrease in happiness and life satisfaction after stepping into the caregiving role. The rest of the indicators, except for contributing to other people’s happiness, displayed negative coefficients without reaching statistical significance.

### Sensitivity analyses

To ensure the robustness of our findings, we conducted several sensitivity analyses to validate the results obtained in the main analysis. Firstly, given the varying timing of individuals becoming caregivers across different groups, we employed a different DiD method that accounts for multiple treatment periods, as recommended by Callaway and Sant’Anna [52]. Due to the relatively small sample sizes within each group, we aggregated the group-time average effects. This involved conducting separate analyses for individuals who became new caregivers in t2, t3, and t4, while consolidating the results. The forest plot presented in S-Fig. 4 displays the estimates obtained from this analysis. Overall, this analysis provides support for the main analysis results, indicating that new caregivers report adverse levels of well-being in general. In this analysis both life satisfaction and engagement are negative and significant. Happiness did not yield significant findings in this analysis but were nonetheless negative and had a relatively similar coefficient to the main analysis (-0.17). Notably, stepping into the caregiving role was associated with lower psychosocial well-being on all indicators in the sensitivity analysis.

Furthermore, recognizing that the main analysis involved imputed values for missing data, we also conducted a sensitivity analysis using complete case data. For this analysis, we included only the 3,915 (142 caregivers and 3,773 non-caregivers) individuals who had non-missing values on all outcome variables. The results obtained from this sensitivity analysis aligned closely with the main findings, reinforcing the robustness and consistency of our findings across different analytical approaches (S-Fig. 5). The only notable difference was that happiness was no longer statistically significant, while loneliness was negative and significant.

Finally, we compared new caregivers with stationary caregivers and ceasing caregivers. New caregivers scored significantly worse on happiness and life satisfaction, compared to stationary caregivers (S-Fig. 6); and significantly worse on worriedness compared to ceasing caregivers (S-Fig. 7). Albeit not significant, new caregivers scored better on contributing to other’s happiness and perceiving life as meaningful, compared to ceasing caregivers.

### Sub-group analyses

To explore potential variations in the impact of caregiving on psychosocial well-being based on age and gender, we conducted sub-group analyses stratified by these variables. We find generally similar patterns between males and females across most outcome measures (Table 2). However, some differences emerge. Becoming a caregiver appeared to increase loneliness and worriedness, and decrease life satisfaction among male caregivers, while no such associations were observed among female caregivers. Conversely, the results indicated that transitioning into a caregiving role was associated with less happiness among women, but not among men. Caregiving also tends to increase the sense of contributing to other people’s happiness among men, but not among women. However, these latter findings were not significant.

**Table 2:** Difference-in-difference estimates

	Overall	By gender		By age			By intensity			By residential status	
*Outcome*		*Male*	*Female*	*18–39*	*40–59*	*60+*	*Daily*	*Weekly*	*Monthly*	*Inside hh*	*Outside hh*
**Negative hedonic well-being**											
Anxious	-0.12 (-0.36, 0.11)	-0.16 (-0.48, 0.17)	-0.09 (-0.43, 0.25)	0.59 (-0.38, 1.56)	-0.07 (-0.51, 0.38)	-0.23 (-0.56, 0.09)	-1.28** (-2.07, -0.48)	0.20 (-0.25, 0.64)	-0.13 (-0.48, 0.22)	-0.32 (-0.77, 0.14)	-0.05 (-0.33, 0.22)
Sadness	-0.21 (-0.45, 0.02)	-0.28 (-0.61, 0.05)	-0.15 (-0.49, 0.19)	0.20 (-0.79, 1.20)	-0.35 (-0.80, 0.09)	-0.28 (-0.60, 0.05)	-0.83** (-1.63, -0.04)	-0.12 (-0.57, 0.33)	-0.17 (-0.52, 0.18)	-0.13 (-0.59, 0.32)	-0.24 (-0.52, 0.03)
Worriedness	-0.24 (-0.48, 0.01)	-0.35** (-0.70, -0.00)	-0.13 (-0.49, 0.23)	-0.11 (-1.09, 0.87)	-0.11 (-0.58, 0.36)	-0.42** (-0.77, -0.07)	-1.05** (-1.89, -0.22)	-0.02 (-0.49, 0.45)	-0.26 (-0.63, 0.11)	-0.11 (-0.59, 0.37)	-0.28 (-0.57, 0.01)
**Positive hedonic well-being**											
Happiness	-0.24** (-0.41, -0.07)	-0.19 (-0.43, 0.05)	-0.28** (-0.53, -0.04)	-0.41 (-1.15, 0.32)	-0.18 (-0.51, 0.14)	-0.18 (-0.41, 0.06)	-1.06** (-1.63, -0.48)	-0.24 (-0.57, 0.08)	-0.15 (-0.41, 0.10)	-0.57** (-0.89, -0.24)	-0.12 (-0.32, 0.08)
Life satisfaction	-0.22** (-0.37, -0.06)	-0.32** (-0.54, -0.10)	-0.12 (-0.34, 0.11)	-0.62 (-1.29, 0.06)	0.05 (-0.25, 0.34)	-0.32** (-0.53, -0.10)	-1.26** (-1.78, -0.74)	-0.07 (-0.37, 0.22)	-0.19 (-0.42, 0.04)	-0.50** (-0.79, -0.20)	-0.12 (-0.30, 0.07)
**Eudaimonic well-being**											
Contributing to others’ happiness	0.02 (-0.15, 0.20)	0.17 (-0.09, 0.42)	-0.11 (-0.35, 0.13)	-0.22 (-0.87, 0.44)	-0.04 (-0.35, 0.28)	0.16 (-0.08, 0.41)	-0.33 (-0.91, 0.25)	0.22 (-0.10, 0.55)	-0.01 (-0.26, 0.25)	-0.19 (-0.52, 0.14)	0.10 (-0.10, 0.30)
Engaged	-0.07 (-0.28, 0.13)	-0.08 (-0.37, 0.20)	-0.07 (-0.35, 0.25)	-0.29 (-1.16, 0.58)	-0.13 (-0.51, 0.26)	-0.00 (-0.27, 0.27)	-0.77** (-1.44, 0.09)	-0.14 (-0.52, 0.24)	0.02 (-0.28, 0.32)	-0.26 (-0.65, 0.12)	-0.01 (-0.24, 0.23)
Meaningful	-0.08 (-0.23, 0.07)	-0.10 (-0.31, 0.11)	-0.05 (-0.27, 0.16)	0.12 (-0.58, 0.82)	0.00 (-0.28, 0.29)	-0.09 (-0.29, 0.12)	-0.79** (-1.30, -0.28)	0.08 (-0.20, 0.37)	-0.03 (-0.25, 0.19)	-0.28 (-0.57, 0.01)	-0.00 (-0.18, 0.17)
**Social well-being**											
Strong social relations	-0.10 (-0.30, 0.10)	-0.03 (-0.34, 0.27)	-0.12 (-0.43, 0.11)	-0.29 (-1.03, 0.45)	0.07 (-0.30, 0.44)	-0.05 (-0.33, 0.24)	-0.92** (-1.60, -0.24)	0.20 (-0.18, 0.58)	-0.16 (-0.46, 0.13)	-0.06 (-0.45, 0.33)	-0.12 (-0.35, 0.12)
Loneliness	-0.16 (-0.39, 0.07)	-0.33** (-0.63, -0.02)	-0.00 (-0.33, 0.32)	-0.20 (-1.18, 0.77)	-0.06 (-0.49, 0.36)	-0.20 (-0.50, 0.11)	-0.80** (-1.55, -0.04)	-0.00 (-0.42, 0.42)	-0.05 (-0.39, 0.28)	-0.36 (-0.28, 0.07)	-0.09 (-0.35, 0.17)

Note: Table shows difference-in-differences estimates adjusted for age (not in analysis stratified by age), gender (not in analysis stratified by gender), education, partner status, and county. All estimates are for new caregivers compared with their non-caregiving counterparts. Inside hh means care recipient live inside the same household. Outside hh means outside the same household

When stratifying the analysis by age groups (18–39 years, 40–59 years, and 60 + years) we find that point estimates of caregivers in the youngest group score worse on 6 of the 10 indicators (Table 2). However, due to few caregivers in this age group, this finding is associated with a large degree of uncertainty. We also note that caregivers in the oldest group score significantly worse on life satisfaction and worriedness compared to non-caregivers. Caregivers aged 40–59 years do not score significantly worse than non-caregivers on any indicators, implying that caregiving responsibilities may have a less distinct impact on the psychosocial well-being of individuals in this age bracket.

Furthermore, we stratified the sample based on caregiving frequency, which includes daily, weekly, and monthly care (Table 2). Distinct patterns emerge. Individuals providing daily care score worse than non-caregivers on all indicators except for contributing to other people’s happiness, which does not show a significant difference. On the other hand, those engaged in caregiving activities on a weekly or monthly basis do not display significant variations in their scores on any of the indicators compared to non-caregivers.

Finally, caregiving within the household has a more pronounced negative impact on psychosocial well-being compared to providing care outside the household (Table 2). Similar to the main analysis, caregiving within the household is associated with lower levels of happiness and life satisfaction. In contrast, we observe no significant changes for individuals providing care outside the household.

## Discussion

### Main findings

In this study we have investigated the impact of entering the caregiving role on individuals’ psychosocial well-being. By examining ten items covering positive and negative hedonic well-being, eudaimonic well-being, and social well-being, we aimed to shed light on the multifaceted negative and positive experiences and perceptions of new caregivers compared to non-caregivers.

Our results indicate that new caregivers generally experience a decline in psychosocial well-being compared to those not engaged in caregiving. This is in line with previous studies focusing on transitionary effects [35, 41, 43, 53]. Increased responsibilities and demands associated with caregiving, coupled with potential emotional and physical strain, may contribute to these declines. Our findings also suggest that the decline in well-being begins prior to the caregiving transition, possibly reflecting worries and concerns about the care recipient’s deteriorating health. Examples of this were also found in Lacey et al.’s [41] recent transitionary paper. In their work, the authors connect these declines to the initiation of a caregiving role when the caregiver has not yet recognized themselves as a caregiver, or when the caregiver is affected negatively by the illness of the care recipient, especially if this is a close family member.

However, while hedonic and social well-being items showed unfavourable trends, indications suggest that new caregivers might perceive themselves as contributing more to the happiness of others, although this was not significant. This notion highlights the potential rewards of caregiving, suggesting that “being there” for a loved one in need can enhance feelings of self-satisfaction and fulfilment [54]. Additionally, it supports Pendergrass et al.’s [32] findings that positive and negative aspects of caregiving can co-occur at the same time. Recognizing the positive aspects and personal growth that can emerge from the caregiving experience is vital in promoting a more holistic understanding of the caregiver’s role. By acknowledging their contribution to the well-being of others, caregivers may find additional sources of motivation and fulfilment in their life.

The level of “strains and gains” associated with caregiving depends on its frequency and intensity. We find larger adverse impacts across all indicators when care is provided daily. Not surprisingly, the only exception was for the impact on perceived contribution to other people’s happiness, which is invariant across frequency of care. Point estimates for daily caregiving were also lower than those for weekly and monthly caregiving, and in supplementary analyses we find that they score significantly lower than those providing care weekly or monthly for most indicators (S-Fig. 8). Other studies have defined caregiving as providing care at least weekly [23]. While we use monthly, particularly for pragmatic power-related reasons, we see that higher intensity and frequency are associated with adverse well-being levels. Similarly, in a literature review [55], authors found that high-intensity caregiving (more than 100 h per month), was associated with adverse outcomes. This suggests the significance of balancing caregiving responsibilities with personal time and self-care activities. Encouraging caregivers to seek respite and assistance, particularly when assuming daily caregiving duties, may contribute to their overall well-being and prevent burnout. We also find adverse results for persons providing care within the household, vs. outside the household. Without adjusting for caregiving frequency, this may also be linked to the intensity of caregiving, or whether there was a perceived choice in taking on the caregiving role [55]. This could also be linked to the relationship between the caregiver and their care recipient. Other studies have found that relationship matters [28–29]. Caring for one’s partner or parent, may be more time-consuming and challenging than caring for a neighbour or a parent in-law [28].

In contrast to previous studies, we found small differences, but mostly uniform trends when stratifying analyses by male and female caregivers. While previous studies [46, 56–58] demonstrate greater emotional distress among female caregivers, we do not find evidence of this in our data. This contrast likely arises because, unlike previous literature which compares male caregivers with female caregivers, we compare male caregivers to male non-caregivers and female caregivers to female non-caregivers. As women tend to score higher on depression and lower on general well-being [58], this could be a result of gender differences unrelated to caregiving in previous studies [59]. Other explanations might be that we did use a monthly cut-off to define caregiving, rather than weekly or daily. Other studies have argued that gender differences could be explained by differences in hours of care provided per week and the number of caregiving responsibilities [12]. Overall, our findings challenge traditional gender roles and highlights the importance of considering individual experiences and circumstances when assessing the effects of caregiving. Understanding the unique challenges faced by caregivers, regardless of gender, can inform the development of tailored support systems and interventions for caregivers.

Finally, when stratifying the analysis by age, we found indications that both younger (18–39 years) and older (60 + years) caregivers were most affected by their new life situation. Yet, the underpinning explanations may vary. Older caregivers may for example face a heavier burden due to physical limitations and the fear of leaving their relative alone after their passing, while younger caregivers may experience depression from balancing social responsibilities with caring for a family member with dementia [12]. Caregivers between 40 and 59 years demonstrated the highest level of stability in their well-being after assuming a caregiving role, which is also the group most frequently being caregivers [2].

### Strengths and weaknesses

One of the key strengths of this study is the utilization of panel data combined with a design allowing for longitudinal tracking of individuals over four waves spanning a two-year period. This quasi-experimental design enables us to capture the changes and trends in well-being experienced by both new caregivers and non-caregivers over time, providing valuable insights into the dynamic nature of caregiving effects. By following individuals for two years, we are able to detect immediate effects and short-term dynamics compared to studies with longer time between the assessments. The short intervals between the surveys are beneficial as most caregiving careers are between two and four years [37–39]. This notion also enhances the relevance and applicability of the findings to real-world caregiving scenarios.

Secondly, we use a robust statistical method (i.e., DiD) to analyse the impact of caregiving on well-being. This strategy serves to mitigate potential confounding factors and provides a more rigorous assessment of the causal relationship between caregiving and psychosocial aspects of life. By employing sound statistical techniques, the study strengthens the validity and reliability of the findings, enhancing the credibility of the conclusions drawn. In addition, both analysis using multiple imputation and complete case analysis yield similar results, as did a traditional DiD alongside a DiD with variation in treatment timing, which enhances the robustness of the findings. However, the relatively small number of data points could yield sub-optimal results in a DiD design, which optimally would have a longer pre-trend [51]. Finally, by incorporating ten items capturing multiple dimensions of psychosocial well-being, we offer a comprehensive and nuanced understanding of the multifaceted effects of caregiving.

Our study also exhibits some weaknesses. All the information gathered in this study is self-reported. This introduces the possibility of biases and subjectivity (e.g., recall and social desirability biases) in the data. As caregiving was only measured in the final survey round, respondents were dependent on remembering back as far as two years. However, given the substantial nature of caregiving responsibilities, and our somewhat crude measure of caregiving intensity (e.g., daily versus weekly), we expect the retrospective accounts to be quite reliable.

Moreover, data were collected during covid-19. Analyses with the same data show that well-being declined following the covid-19 pandemic [23], and the evolving circumstances may have impacted participants’ responses and overall survey dynamics. To account for this, we have adjusted for time trends. Still, caregiving during covid-19 might have been particularly challenging and one could expect that more people would become caregivers when respite and nursing homes closed. However, t4 was the timepoint when most people said they became caregivers.

A final weakness is that our study suffers from considerable attrition observed throughout the data collection process. Attrition can compromise the internal and external validity of the findings, as the characteristics of individuals who drop out may differ from those who remain in the study. Of note, because caregiving was only measured in the final wave, individuals participating in only prior waves could not be included. Attrition analysis (S-Table 2) shows that 75% of those answering the questionnaire in wave 1, did not make it until wave 4. Subsequent dropouts also scored significantly lower on all indicators (except contributing to other people’s happiness) in wave 1, compared to those included in this study. This suggests that individuals in our sample have a higher well-being compared to the general population, indicating that we might be underestimating the negative effect of caregiving.

## Conclusion

In conclusion, our study reveals that transitioning into a caregiving role may have broad, mostly negative effects on caregivers’ well-being. Typically, new caregivers experience a decline in life satisfaction and happiness, along with heightened psychological distress and loneliness. Notably, the impact on eudaimonic aspects of well-being was less pronounced, and in some instances, modestly beneficial. There was also significant heterogeneity in these effects. The adverse impacts were more pronounced among those providing the most intensive care. Differences in effects based on gender were minimal.

Improved understanding of the dynamic and multifaceted nature of the caregiving experience is potentially of great value both to future and current caregiver, caregiver support groups, researchers, public planners, and other stakeholders. Our findings highlight the nuanced dynamics of caregiving and the need for targeted interventions and support systems to mitigate the potential negative consequences associated with caregiving roles. Further research should aim to explore the underlying mechanisms, specifically delving into whether negative emotions stem from the responsibilities associated with caregiving or are primarily driven by the illness of a family member. Future research is also needed to explore, design, and clarify specific measures to help promote and support well-being among carer groups.

### Electronic supplementary material

Below is the link to the electronic supplementary material.

Supplementary Material 1

## Data Availability

Data are not owned by the authors but can be applied for through the website helsedata.no/en. Stata and R-code are available upon request to the corresponding author.

## Footnotes

1 I actively contribute to the happiness and quality of life of others.

2 All in all, to what extent do you experience that what you do in life is meaningful?

3 My social relationships are supportive and rewarding.

## Abbreviations

DiD: Difference–in–differences
Hh: Household
NCPHS: Norwegian Counties Public Health Survey
RR: Response rate
t1/t2/t3/t4: Survey round
