# Supplementary material for: The double-edged sword of becoming a caregiver: dynamic impact on four dimensions of well-being in Norway
Source: BMC Psychol. 2024 Mar 4;12:120. doi: 10.1186/s40359-024-01623-x (PMC10913458; doi:10.1186/s40359-024-01623-x)
Supplement: Supplementary file 1 — Supplementary Material 1 [file 40359_2024_1623_MOESM1_ESM.pdf]

Supplementary file for the paper

The Double-Edged Sword of Becoming a Caregiver: Dynamic Impact on Four Dimensions of Well-Being in Norway

(Short titles)

Supplementary Table 1: Survey questions (exact phrasing). p. 2

Supplementary Fig 1: Correlation plot of well-being dimensions. p. 2

Supplementary Fig 2: Time of becoming caregiver. p. 3

Supplementary Fig 2: Trajectories of new caregivers and non-caregivers. p. 4

Supplementary Fig 4: Callaway & Sant'Anna. p. 5

Supplementary Fig 5: Complete case analysis. p. 6

Supplementary Fig 6: New vs. stationary caregivers. p. 7

Supplementary Fig 7: New vs. ceasing caregivers. p. 7

Supplementary Fig 8: Daily caregiving compared to weekly or monthly caregiving. p. 8

Supplementary Table 2: Attrition analysis. p. 9

Supplementary Table 1: Survey questions (exact phrasing).

Question	Short name	Scale	Positive or negative
Think about how you have felt in the past 7 days. To what extent were you...			
... anxious?	Anxious	0 – 10	Negative
... engaged?	Engaged	0 – 10	Positive
... happy?	Happiness	0 – 10	Positive
... lonely?	Loneliness	0 – 10	Negative
... down and sad?	Sadness	0 – 10	Negative
... worried?	Worriedness	0 – 10	Negative
Overall, to what extent do you experience that what you do in life is meaningful?	Meaningful	0 – 10	Positive
Overall, how satisfied are you with your life at the moment?	Life satisfaction	0 – 10	Positive
How much do you agree with the statements below?			
My social relationships are supportive and rewarding.	Strong social relations	0 – 10	Positive
I actively contribute to the happiness and quality of life of others.	Contributing to other's happiness	0 – 10	Positive

Supplementary Figure 1: Correlation plot of well-being dimensions.

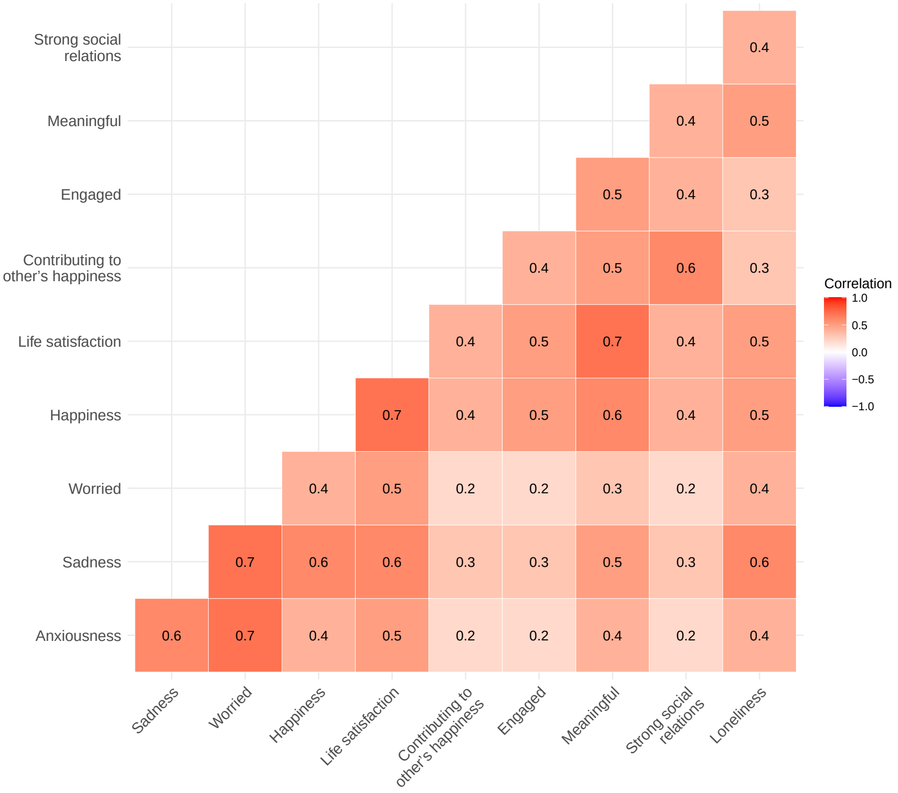

Note: All correlations are significant ($p < 0.05$).

Supplementary Figure 2: Time of becoming caregiver.

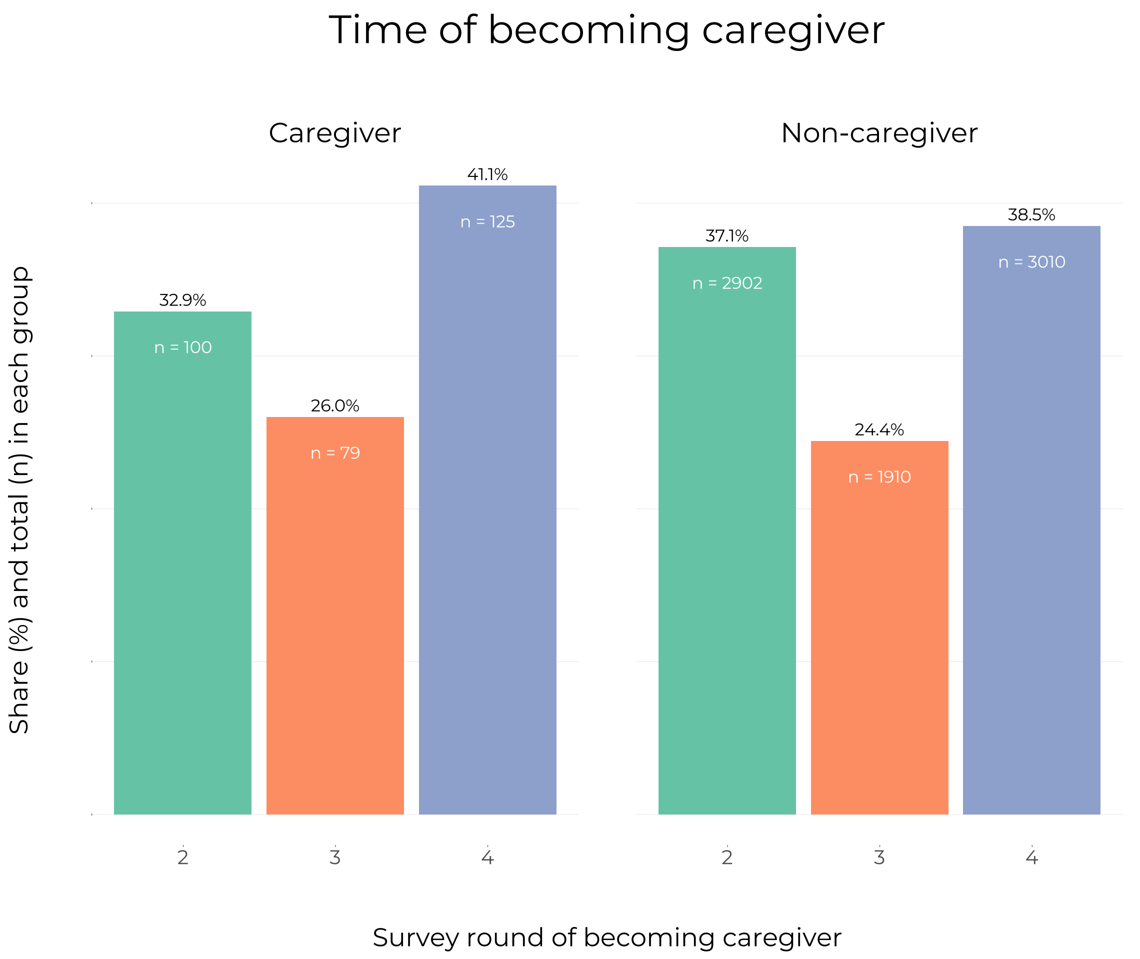

Supplementary Figure 3: Trajectories of new caregivers and non-caregivers.

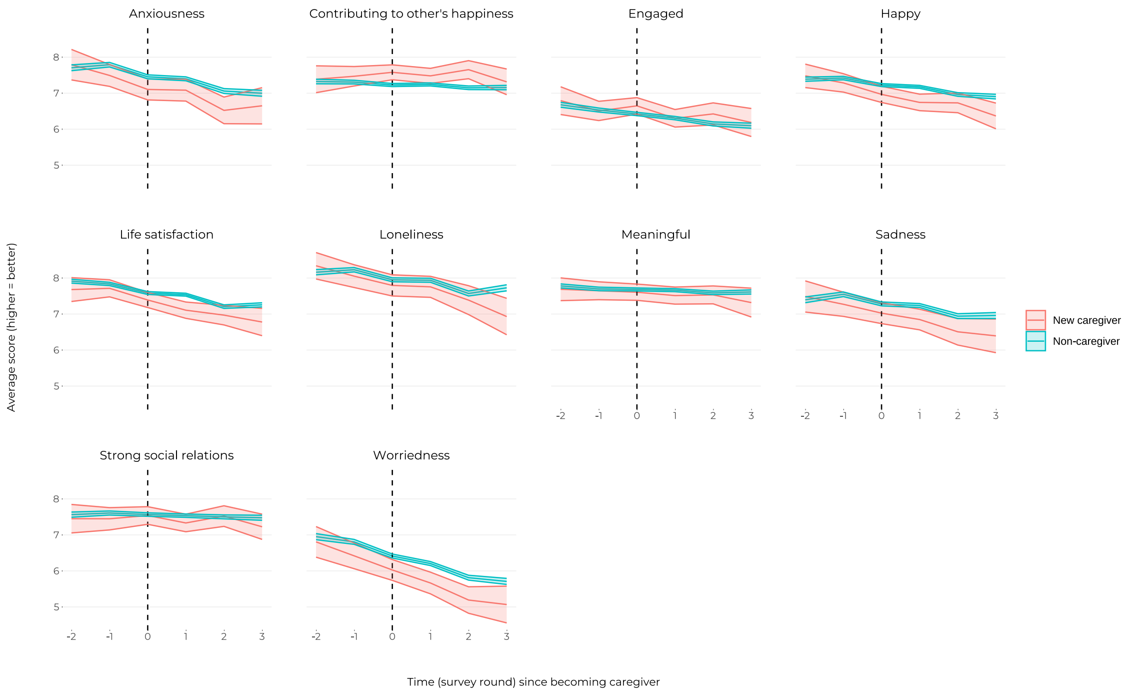

Supplementary Figure 4: Callaway & Sant'Anna.

Outcome	DiD (95% CI)
Anxiousness	-0.02 (-0.29 to 0.24)
Sadness	-0.17 (-0.45 to 0.10)
Worriedness	-0.22 (-0.54 to 0.09)
Happiness	-0.17 (-0.38 to 0.03)
Life satisfaction	-0.23 (-0.43 to -0.03)
Contributing to other's happiness	-0.07 (-0.25 to 0.12)
Engaged	-0.22 (-0.43 to -0.00)
Meaningful	-0.07 (-0.27 to 0.13)
Strong social relations	-0.16 (-0.37 to 0.05)
Loneliness	-0.13 (-0.40 to 0.14)

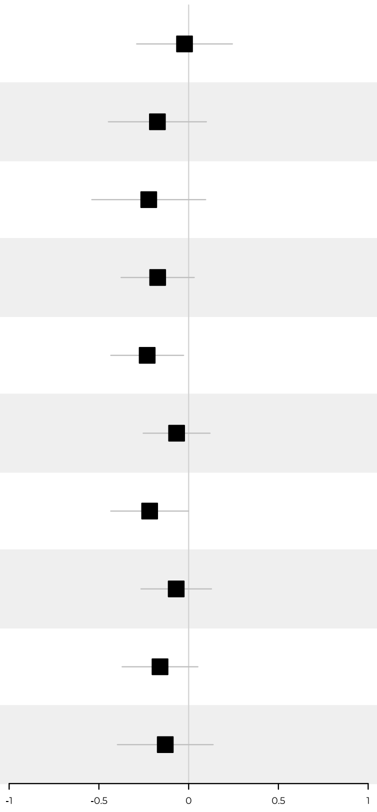

Note: The plot visualise the difference-in-difference estimates with 95% confidence intervals for new caregivers compared to non-caregivers. Estimates were calculated as the aggregate group-time average effects as proposed by Callaway & Sant'Anna (2021) and adjusted for gender, age, education, partner status, and county.

Supplementary Figure 5: Complete case analysis.

Outcome	DiD (95% CI)
Anxiousness	-0.19 (-0.52 to 0.14)
Sadness	-0.33 (-0.67 to 0.02)
Worriedness	-0.20 (-0.55 to 0.16)
Happiness	-0.19 (-0.43 to 0.05)
Life satisfaction	-0.39 (-0.61 to -0.17)
Contributing to other's happiness	0.08 (-0.16 to 0.33)
Engaged	-0.22 (-0.50 to 0.07)
Meaningful	-0.20 (-0.41 to 0.01)
Strong social relations	-0.13 (-0.41 to 0.15)
Loneliness	-0.34 (-0.66 to -0.02)

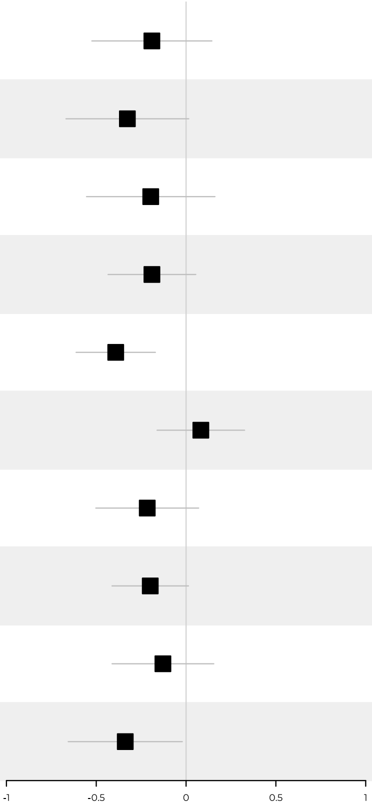

Note: The plot visualise the difference-in-difference estimates with 95% confidence intervals for new caregivers compared to non-caregivers, using only the 3,915 individuals with complete data on all outcomes.

Supplementary Figure 6: New vs. stationary caregivers.

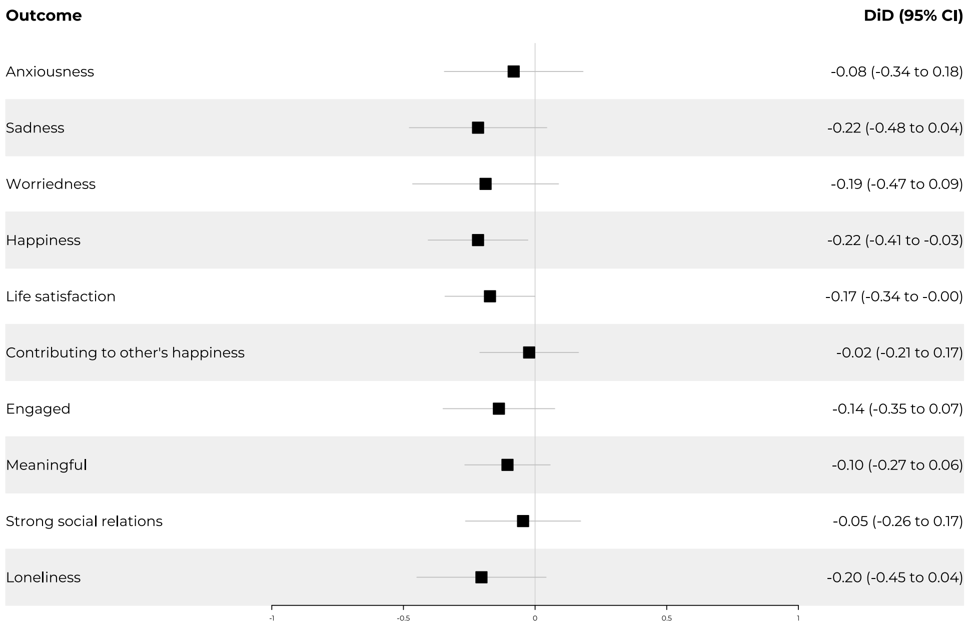

Supplementary Figure 7: New vs. ceasing caregivers.

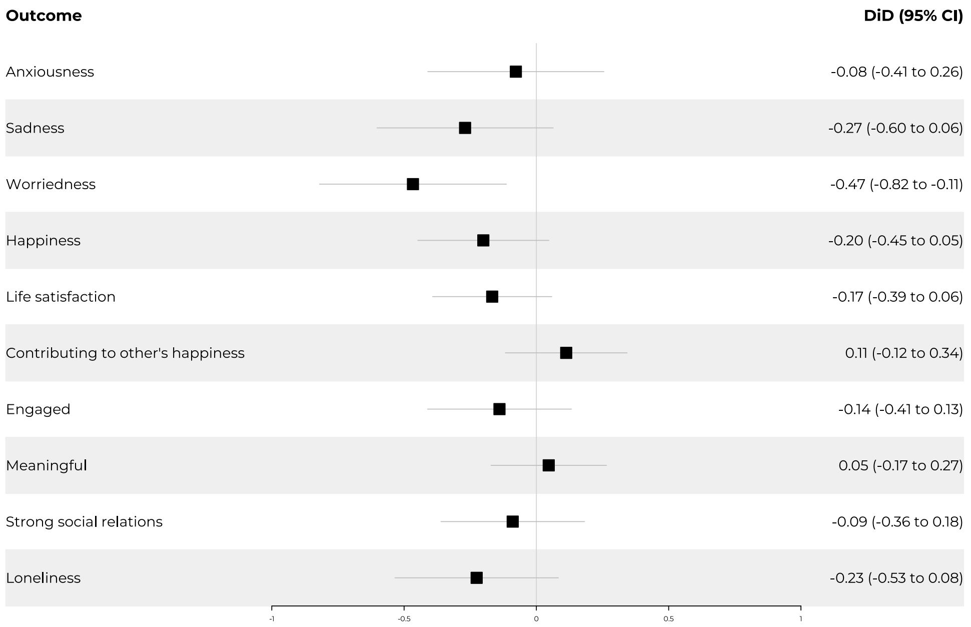

Supplementary Figure 8: Daily caregiving compared to weekly or monthly caregiving.

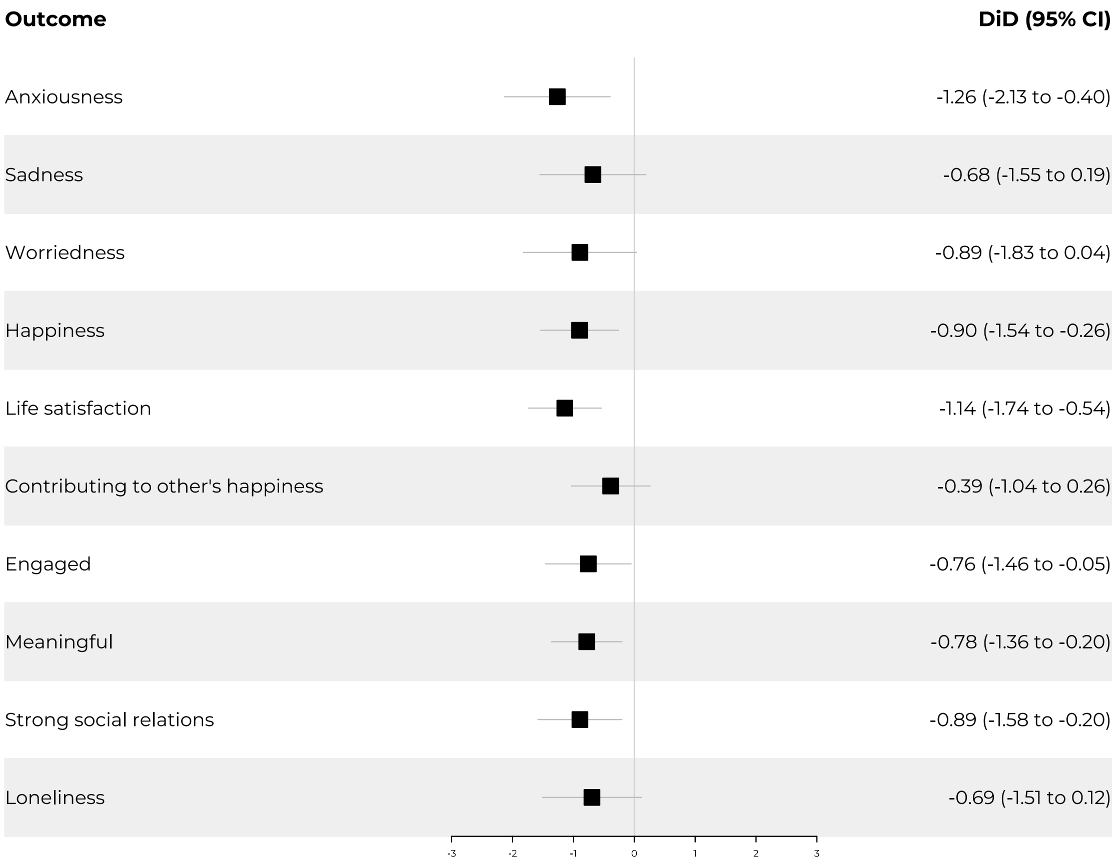

Supplementary Table 2: Attrition analysis.

	Non-incl	Incl.	Mean (non-incl)	Mean (incl.)	Diff.	St. err.	T-value	P-value
Anxiousness, t1	41760	8686	7.173	7.534	-.361	.031	-11.70	0
Anxiousness, t2	3673	6642	7.388	7.824	-.436	.051	-8.60	0
Anxiousness, t3	3035	6483	6.725	7.176	-.451	.058	-7.80	0
Sadness, t1	41757	8685	6.978	7.286	-.308	.031	-10.00	0
Sadness, t2	3658	6593	7.223	7.610	-.387	.052	-7.50	0
Sadness, t3	3034	6425	6.600	7.041	-.441	.058	-7.70	0
Worriedness, t1	41785	8687	6.397	6.705	-.308	.032	-9.85	0
Worriedness, t2	3664	6628	6.268	6.644	-.376	.053	-7.05	0
Worriedness, t3	3063	6504	5.683	6.003	-.321	.057	-5.70	0
Happiness, t1	41866	8704	7.090	7.292	-.202	.024	-8.40	0
Happiness, t2	3777	6756	7.229	7.406	-.176	.039	-4.55	0
Happiness, t3	3088	6565	6.756	7.030	-.275	.044	-6.35	0
Life satisfaction, t1	41895	8708	7.492	7.742	-.250	.024	-10.55	0
Life satisfaction, t2	3830	6831	7.445	7.793	-.347	.038	-9.05	0
Life satisfaction, t3	3129	6611	6.974	7.317	-.344	.043	-8.15	0
C. other's happiness, t1	41836	8704	7.300	7.309	-.009	.025	-.350	.722
C. other's happiness, t2	3798	6805	7.284	7.313	-.029	.040	-.700	.472
C. other's happiness, t3	3095	6584	6.996	7.139	-.142	.044	-3.25	.001
Engaged, t1	41724	8679	6.376	6.561	-.185	.026	-7.10	0
Engaged, t2	3619	6563	6.197	6.481	-.284	.046	-6.15	0
Engaged, t3	2991	6393	5.992	6.303	-.311	.049	-6.35	0
Meaningful, t1	41866	8701	7.477	7.733	-.257	.026	-10.15	0
Meaningful, t2	3792	6779	7.327	7.675	-.348	.043	-8.20	0
Meaningful, t3	3111	6590	7.318	7.673	-.355	.044	-8.20	0
Strong social rel., t1	41799	8693	7.356	7.537	-.182	.029	-6.45	0
Strong social rel., t2	3827	6838	7.438	7.608	-.171	.044	-3.85	0
Strong social rel., t3	3114	6591	7.296	7.551	-.255	.049	-5.25	0
Loneliness, t1	41776	8699	7.732	8.063	-.332	.031	-10.7	0
Loneliness, t2	3653	6568	7.934	8.242	-.308	.053	-5.85	0
Loneliness, t3	3012	6410	7.157	7.670	-.512	.063	-8.20	0

Note: Table shows differences in mean scores between those not included (due to attrition) and those included in the study sample. Negative values in differences indicate that lost persons score worse than included persons. P-value indicate significance on 0.05-level.
